# Resistance Exercise Training as a Primary Countermeasure to Age-Related Chronic Disease

**DOI:** 10.3389/fphys.2019.00645

**Published:** 2019-06-06

**Authors:** Jonathan C. Mcleod, Tanner Stokes, Stuart M. Phillips

**Affiliations:** Department of Kinesiology, McMaster University, Hamilton, ON, Canada

**Keywords:** aging, sarcopenia, physical mobility, resistance training, chronic disease risk, cardiovascular disease, type 2 diabetes, cancer

## Abstract

Age is a primary risk factor for a number of chronic diseases including mobility disability, cardiovascular disease (CVD), type 2 diabetes (T2D), and cancer. Most physical activity guidelines emphasize the performance of 150 min of moderate-to-vigorous or 75 min of vigorous aerobic exercise training (AET) weekly for reduction of chronic disease risk. Nonetheless, there is an emerging body of evidence showing that resistance exercise training (RET) appears to be as effective as AET in reducing risk of several chronic diseases. It may also be that RET is more effective than AET in some regards; the converse is likely also true. We posit that the perceived divergent exercise mode-dependent health benefits of AET and RET are likely small in most cases. In this short review, our aim is to examine evidence of associations between the performance of RET and chronic health disease risk (mobility disability, T2D, CVD, cancer). We also postulate on how RET may be influencing chronic disease risk and how it is a critical component for healthy aging. Accumulating evidence points to RET as a potent and robust preventive strategy against a number of chronic diseases traditionally associated with the performance of AET, but evidence favors RET as a potent countermeasure against declines in mobility. On the basis of this review we propose that the promotion of RET should assume a more prominent position in exercise guidelines particularly for older persons.

## Introduction

Cardiovascular disease (CVD), cancer, and type 2 diabetes (T2D) are leading causes of morbidity and mortality in older adults aged 65 years and older (Roth et al., 2015; Tanday, 2016). Aging is also the single biggest predictor for mobility impairments, which can exacerbate the risk for all of the aforementioned chronic diseases (Newman et al., 2006). Pharmacological agents are frequently prescribed to treat or delay the progression of major chronic diseases in mobility-impaired older individuals; however, most if not all of these therapies have some degree of off-target effects that may be undesirable or reduce compliance with prescribed dosing. Global population aging has resulted in a concomitant increase of people living with age-related chronic disease and also with impaired physical mobility. Low cost, widely implementable multi-condition pharmaceutical interventions that have a low side-effect profile and mitigate risk for all common chronic diseases while alleviating the risk of mobility decline do not presently exist. However, routine exercise can variably mitigate the age-related reduction in physical mobility and reduce chronic disease risk to an appreciable extent.

The progressive decline of skeletal muscle mass and strength with aging is collectively referred to as sarcopenia, and is prognostic for mobility disability (Visser et al., 2002, 2005) and chronic disease risk (Pedersen and Saltin, 2015). Regular physical activity (defined here as any bodily movement produced by the contraction of skeletal muscle that increases energy expenditure; Caspersen et al., 1985) and exercise (physical activity that is planned, structured, and repetitive; Caspersen et al., 1985) are cornerstones in the primary prevention of chronic diseases (Pedersen and Saltin, 2015) and also for mitigating risk of mobility disability in older persons (Pahor et al., 2014; Villareal et al., 2017).

Resistance exercise (RE) and aerobic exercise (AE) are modalities of exercise that are traditionally conceptualized as existing on opposite ends of an exercise continuum in terms of the phenotypes they lead to. A common misconception is that RE training (RET) and AE training (AET) also result in separate health benefits, but we propose this is an artifact of the greater volume of data that currently exists for AET as opposed to RET. Currently, most physical activity guidelines advise, as their primary message, that older adults should perform at least 150 min of moderate-to-vigorous or 75 min of vigorous AET weekly for the reduction of chronic disease risk and maintenance of functional abilities (American College of Sports Medicine, 2009; Canadian Society for Exercise Physiology, 2011; American Heart Association, 2018; Piercy et al., 2018). However, there is an emerging body of evidence to suggest that RET can be as effective as AET in reducing chronic disease risk and is particularly potent for maintaining mobility in older adults (Tanasescu et al., 2002; de Vries et al., 2012; Grontved et al., 2012; Stamatakis et al., 2018).

The aim of this review is to provide an up-to-date evidence-based narrative review of the efficacy of RET in combating chronic health disease (mobility disability, T2D, CVD, and cancer) risk in older adults. To achieve this aim, we summarize data derived predominantly from humans, but will draw upon important findings from preclinical disease models to substantiate our arguments and provide additional mechanistic insight not available in human observational trials.

## Resistance Exercise Training and Physical Mobility

Mounting evidence from systematic reviews (Theou et al., 2011), meta-analyses (de Vries et al., 2012; Gine-Garriga et al., 2014; de Labra et al., 2015), and umbrella reviews (Jadczak et al., 2018) convincingly show that exercise interventions combining RET and AET are the most effective for combating age-related declines in physical mobility. Villareal et al. (2017) demonstrated that obese older adults with mobility limitations who performed combined (AET and RET) training improved objective and subjective measures of functional ability more than individuals randomized to either RET or AET alone. However, as is often the case in these clinical trials, the combined RET plus AET group performed a larger volume of exercise than the groups performing either modality alone, which likely confounded the results.

A recent umbrella review demonstrated that RET in pre-frail and frail older adults could significantly enhance muscular strength, gait speed, and physical performance (Jadczak et al., 2018). Pooled data from 33 randomized controlled trials showed that performing RET resulted in a statistically significant improvement in physical function (Liu and Latham, 2009). de Vries et al. (2012) have argued that RET is of greater importance in an exercise program than AET for improving physical mobility in community-dwelling, mobility-impaired older adults. On the contrary, a recent meta-analysis conducted by Hortobagyi et al. (2015) found similar improvements in gait speed in healthy older adults performing either AET or RET. The heterogeneity in experimental design across studies (i.e., participant characteristics, training variables [frequency, intensity, time]), and methods used to assess mobility, can make it difficult to conclude which exercise modality is most efficacious in combatting mobility declines in older adults. Cognizant of this limitation, future randomized controlled trials are warranted to investigate which exercise mode is most effective in improving physical function in older adults. Nevertheless, when looked at collectively, the evidence suggests that RET can play a fundamental role in improving and/or maintaining functional mobility that is at least on par with those imbued by AET, in older adults.

The underlying mechanisms by which RET attenuates the decline in physical function of older adults is likely multifaceted. However, low muscle mass and strength are associated with poor physical function (Visser et al., 2002), and predictive of future mobility impairment in older adults (Visser et al., 2005). A recent cross-sectional analysis determined that community-dwelling older adults with low muscle mass and combined low muscle mass and function had a 1.6- and 12.2-increased odds of being physically dependent, respectively (Dos Santos et al., 2017). RET is a potent stimulus for skeletal muscle hypertrophy and augmenting strength in older adults. Indeed, a meta-analysis containing 49 randomized controlled trials concluded that after an average of 20.5 weeks of RET, older adults gained 1.1 kg of lean body mass (Peterson et al., 2011). Moreover, RET (either alone or as part of a combined training program) enhanced strength gains in frail older adults more than combined exercise programs without RET. Whole-body progressive RET (2 sets of 65–85% of 1 repetition maximum [1RM]) 3 times a week for 6 months attenuated losses in bone mineral density, lean mass, and muscular strength in obese frail participants to a greater extent than combined training or AET (jogging/running for 60 min at 65–85% of heart rate peak [HR_peak_]; Villareal et al., 2017). In contrast, AET alone is ineffective at inducing comparable increases in skeletal muscle mass and strength (Grgic et al., 2018). In addition, RET can improve neurological (i.e., increased central motor drive, elevated motoneuron excitability; Aagaard et al., 2002), psychological (i.e., self-efficacy; Kekalainen et al., 2018), and/or cardiovascular function (i.e., maximal stroke volume; Roberson et al., 2018) – all of which have been hypothesized to contribute to skeletal muscle performance in older adults (Tieland et al., 2018). Thus, it is not surprising that RET exerts beneficial effects on physical function in older adults – regardless of whether muscle hypertrophy is observed – through factors extrinsic to skeletal muscle. Further work is needed to identify the dominant mechanism by which RET can combat mobility impairments.

Although high-intensity RET (≥70% of 1RM) is generally more effective than low-to-moderate intensity RET (30–69% of 1RM) in combating mobility decrements (de Vries et al., 2012), the heterogeneity between studies makes it is difficult to conclude with a high degree of certainty the optimal RET intensity. It should be noted that RET where one’s own body weight is used for resistance and in which activities of daily living are simulated (i.e., body-weight squat) can improve indices of physical function in older adults to a similar extent as conventional RET (requiring external loads; Lustosa et al., 2011). Notwithstanding, multicomponent exercise programs (consisting of RET, AET, and balance training in combination) appear to be the best strategy for attenuating declines in physical mobility (Theou et al., 2011; de Vries et al., 2012; Cadore et al., 2013; Gine-Garriga et al., 2014; de Labra et al., 2015; Jadczak et al., 2018).

## Resistance Exercise Training and Type 2 Diabetes

A hallmark of aging is the progressive deterioration of whole-body insulin sensitivity and consequent impairment of glycemic control (Jackson et al., 1982) that predispose older adults to T2D. T2D is the nexus of insulin resistance and impaired β- cell function (Weyer et al., 2001), and is one of the most prevalent metabolic diseases afflicting older adults (Statistics Canada, 2016). In older adults, the insulin-mediated suppression of hepatic glucose output is delayed and peripheral glucose uptake into skeletal muscle is impaired (Jackson et al., 1982). Inevitably, the inability of the aging pancreas to produce and secrete enough insulin to buffer the resistance in peripheral and hepatic tissues leads to T2D.

Given that ~80% of glucose is deposited in skeletal muscle during postprandial periods (Thiebaud et al., 1982), the loss of muscle mass and that of muscle metabolic quality with advancing age are thought to be primary drivers of insulin resistance and T2D development in older adults (DeFronzo and Tripathy, 2009). Epidemiological data (Srikanthan and Karlamangla, 2011) demonstrate an inverse relationship between lean body mass and insulin resistance, an effect that appears independent of, but exacerbated by, obesity in older adults (Srikanthan et al., 2010). Moreover, declining muscle strength and progressive mobility impairment with age likely cause a reduction in daily physical activity, which alone is sufficient to induce metabolic dysfunction (McGlory et al., 2018).

Recent work from our laboratory demonstrated that a reduction of habitual daily stepping for a period of 2 weeks (<1,000 steps/day) in prediabetic older adults results in significant impairments in glycemic control and an insulin-resistant state in response to a 75-g oral glucose challenge (McGlory et al., 2018). Importantly, participants failed to recover baseline insulin sensitivity upon returning to habitual activity for 2 weeks. Recently, Reidy et al. (2018) confirmed the induction of insulin resistance following step-reduction using a hyperinsulinemic-euglycemic clamp, the gold-standard method to assess insulin sensitivity. In contrast to our findings, however, Reidy et al. (2018) demonstrated that older adults fully recovered baseline insulin sensitivity following a return to habitual stepping. These data together suggest that reductions in physical activity levels in older adults contribute substantially to the development of insulin resistance that precedes diabetes development.

Lifestyle interventions are arguably the most effective therapeutic strategies in terms of preventing and managing diabetes. Indeed, the Diabetes Prevention Program (DPP) demonstrated that lifestyle modifications (i.e., diet and exercise) were associated with a greater reduction (58 vs. 31%; Knowler et al., 2002) in the incidence of T2D compared to metformin – the current frontline therapy for T2D (American Diabetes Association, 2014). Importantly, however, the DPP focused on AET with little consideration of the beneficial effects of RET on glycemic control. Interesting data from Davy et al. (2017) demonstrated that after only 3 months (2×/week) of progressive, supervised, whole-body RET (1 set at 70–80% 1RM), ~34% of overweight/obese prediabetic older adults achieved normal glucose tolerance. These findings are not isolated and, when considered collectively (Zachwieja et al., 1996; Iglay et al., 2007), support the effectiveness of RET to improve glycemic control in elderly adults. These improvements would be expected, and have been reported to translate into reduced T2D incidence in the elderly (Grontved et al., 2012). Indeed, an analysis of ~32,000 men between the ages of 40–75 years from the Health Professionals’ Study demonstrated that men engaging in at least 150 min/week of RET had a 34% lower risk of developing diabetes over an 18-year period (Grontved et al., 2012). Model-derived estimates predict that a risk reduction of this magnitude (~30%) would save ~$1.5 billion in healthcare expenditure (Bilandzic and Rosella, 2017).

People with diagnosed type 1 and type 2 diabetes can also benefit from the inclusion of RET for the management of glycemia as an adjunct therapy to antidiabetic pharmaceutical agents (American Diabetes Association, 2018). In one study, an acute bout of either AE (running at 60% maximal oxygen uptake [VO_2max_]) or whole-body RE (3 sets at 70% 1RM) resulted in significant reductions of plasma glucose levels in physically active type 1 diabetics (Yardley et al., 2013). Although the decrement was greater during AE, interstitial glucose monitoring post-exercise demonstrated that only the participants performing RE maintained lower plasma glucose levels over the ensuing 24 h. In a recent meta-analysis including 360 older patients with T2D, RET for at least 8 weeks was also associated with clinically relevant improvements in glycated hemoglobin (HbA1c) and muscle strength (Lee et al., 2017). The RET-induced improvement in HbA1c was also observed in 7/8 studies systematically analyzed by Gordon et al. (2009). Future randomized controlled trials are now needed to examine the salient mechanisms driving the rejuvenation of insulin sensitivity in response to RET, which are briefly considered below.

Muscle contraction *per se* improves glucose homeostasis through insulin-dependent and independent signaling pathways (Holloszy, 2005). Theoretically, growth or atrophy of skeletal muscle is expected to perturb glucose handling through expansion or contraction, respectively, of the predominant glucose disposal site; however, RET can improve insulin sensitivity independently of changes in lean body mass (Holten et al., 2004), indicating that intrinsic insulin signaling is improved. After binding to its membrane receptor, insulin initiates a signaling cascade that converges on the phosphorylation of AS160, permitting the translocation and docking of GLUT4 transporters onto the sarcolemma and enhancing glucose uptake. Insulin-mediated phosphorylation of AS160 is impaired in older adults resulting in reduced GLUT4 delivery to the sarcolemma and decreased muscle uptake (Consitt et al., 2013). Once inside the cell, a majority of glucose is directed toward glycogen synthesis in normoglycemic adults *via* glycogen synthase activation. This process is impaired, and is thought to be a primary driver of insulin resistance, in T2D (Shulman et al., 1990; Pedersen et al., 2015). Glycogen synthase content is also reduced in aged skeletal muscle (Pastoris et al., 2000) and, together with reduced GLUT4 translocation, likely contributes to the marked reduction in peripheral glucose disposal in insulin-resistant older adults (Jackson et al., 1988). Fortunately, these age-related impairments are partially reversible with RET. For instance, sedentary older men participating in 8 weeks of combined RET and AET (training variables not published) demonstrated increased skeletal muscle hexokinase II, Akt2, glycogen synthase, and GLUT4 protein content (Bienso et al., 2015). These changes were associated with a significant decrease of insulin area under the curve during an oral glucose tolerance test (OGTT), in the absence of change in glucose area under the curve, indicating an improvement in whole-body insulin sensitivity (Bienso et al., 2015). Finally, older adults participating in RET (3 sets at 60–85% 1RM) for 24 weeks exhibited large increases (~57%) in mitochondrial oxidative capacity (Jubrias et al., 2001), which is likely linked to the training-induced improvements in insulin sensitivity.

We propose that there is good rationale and data in support of a role for RET in the prevention and treatment of insulin resistance in older adults. However, it currently remains unclear which RET training variable is most closely related with the RET-induced improvements in glycemic control in individuals with T2D. Evidence from a systematic review (Gordon et al., 2009) suggests that exercise intensity is the key variable and that performing high-intensity RET (≥70% 1RM) results in the greatest improvement in glycemic control. However, the majority of trials included in this study did not control for the total volume of exercise being performed. Indeed, a recent study in individuals with T2D demonstrated that, when matched for exercise volume, there was no significant difference in glycemic control with high- or low-intensity RET (75 vs. 50% of 1RM, respectively) (Yang et al., 2017). Further work is needed to confirm these results; nonetheless, this work provides rationale that older adults with T2D (or at high risk for developing T2D) should simply concentrate on performing RET without having to worry about the exercise intensity. The resolution of hyperglycemia and hyperinsulinemia in metabolically compromised older adults through exercise not only prevents the pathogenesis of T2D, but also the associated microvascular complications that, if unabated, are precursors to a number of comorbidities in persons with T2D.

## Resistance Exercise Training and Cardiovascular Disease

AET reduces the risk of CVD and mortality (Yusuf et al., 2004; O’Donnell et al., 2016), and as a result has been the focus of lifestyle interventions targeting these ailments. This observation comes as no surprise given that improved cardiorespiratory fitness – a hallmark adaptation in response to AET – is inversely associated with CVD risk and mortality (Nauman et al., 2017). In addition to cardiorespiratory fitness, muscle mass and strength are also independently associated with risk for CVD and mortality (Ruiz et al., 2008; Srikanthan et al., 2016; Kim et al., 2017), and yet RET is usually emphasized far less as an exercise modality that reduces CVD risk.

A follow-up from the Health Professional’s study demonstrated that RET for at least 30 min per week resulted in a similar risk reduction compared to 2.5 h of brisk walking in fatal and nonfatal myocardial infarction (Tanasescu et al., 2002). Similarly, a recent analysis of the Women’s Health study showed that women engaging in 60–120 min of RET per week had a similar 22% reduced risk of incident CVD as women engaging in 60–120 min of AET per week (Shiroma et al., 2017). Smutok et al. (1993) randomized older men at risk for developing CVD to either whole-body, progressive RET (2 sets at 60–70% 1RM) or treadmill walking/jogging (75–85% heart rate reserve) for 20 weeks and found that RET reduced risk factors associated with CVD to a similar degree as walking/jogging on the treadmill. Clearly, the aforementioned evidence suggests that RET is associated with reductions in CVD risk and mortality that are similar in magnitude as those provoked by AET.

From a mechanistic perspective, RET results in favorable improvements in a constellation of risk factors associated with CVD to the same degree as AET (i.e., blood pressure, blood lipids, insulin sensitivity, and vascular function; Yang et al., 2014). Graded increases in systolic blood pressure (SBP) and diastolic blood pressure (DBP) remain two of the most significant modifiable risk factors for CVD (Lopez et al., 2006). Meta-analyses demonstrate that RET induces reductions in SBP and DBP that are of similar or greater magnitude to AET in healthy adults (Cornelissen and Smart, 2013; MacDonald et al., 2016). Notably, the magnitude of RET-induced reductions in SBP (5–6 mmHg) and DBP (3–4 mmHg) are associated with an 18% reduction of major cardiovascular events (Blood Pressure Lowering Treatment Trialists Collaboration, 2014). The beneficial effects of RET on SBP and DBP extend to individuals with hypertension (MacDonald et al., 2016). In fact, compared to individuals with normal blood pressure, individuals with hypertension yield the largest reductions in blood pressure following RET (MacDonald et al., 2016). Considering that reductions in SBP and DBP serve as a cornerstone of CVD prevention in individuals with hypertension (Joseph et al., 2017), RET may serve as an adjunct or even alternative treatment to commonly prescribed antihypertensive medications. Future randomized controlled trials are warranted to compare RET-induced BP reductions to antihypertensive medications in individuals with hypertension.

The above-mentioned benefits of RET on cardiovascular health extend to individuals with T2D (Yang et al., 2014). Considering that compared to nondiabetic individuals, persons with T2D have a two- and fourfold risk of developing CVD, these findings are particularly important (Emerging Risk Factors Collaboration, 2010). Age-specific mortality rates of CVD fell by ~15% between 2005 and 2015 (Roth et al., 2015); however, as a consequence of the rising prevalence of older adults living with T2D, estimates suggest an increasing proportion of cardiovascular mortality may be attributable to this metabolic condition. Although the beneficial effects of RET on cardiovascular health are clear, RET is not typically endorsed as a mode of exercise for reducing CVD risk (American Heart Association, 2018).

Clinical prescription of RET is rare largely due to the perception that AET is safer and likely easier to implement in patients with CVD. It has been suggested that high-pressure loads induced on the heart by RET can lead to a mild form of cardiac hypertrophy, which can lead to higher mortality risk (Kamada et al., 2017). However, excessive elevation of blood pressure is seen only with high-intensity RET (≥70% of 1RM) (MacDougall et al., 1985), and is generally not a concern for lighter-to-moderate intensity RET (30–69% of 1RM). Williams et al. (2007) argue that most RET studies evaluating safety have selected low-risk individuals, and that the studies do not provide reliable estimates of event rates on a population basis. However, this argument has limited supporting evidence. For example, Hollings et al. (2017) pooled together data from 5 studies evaluating adverse events during low-to-moderate intensity RET (30–69% of 1RM) in older adults with CVD, and found that RET was actually associated with a *lower* rate of adverse cardiovascular complications than AET. Furthermore, a meta-analysis in older adults at risk for developing CVD demonstrated that arterial stiffness (a correlate of cardiovascular mortality; Laurent et al., 2001) does not increase or worsen following RET (Evans et al., 2018). In fact, an acute bout of RE appears to be more protective from ischemic changes than a bout of AE, and results in a lower heart rate response and higher diastolic perfusion pressure (Featherstone and Holly, 1993). These physiological changes result in a more favorable supply of oxygen to the myocardium during RE. Thus, the misconception that RET is less safe than AET in physically or metabolically vulnerable individuals lacks empirical evidence.

Our review leads us to propose that there is good evidence supporting a role for RET in maintaining cardiovascular health and again this is likely to be of a comparable magnitude in terms of risk reduction as that seen with AET. Regarding the exercise intensity required to exert beneficial effects on CVD risk factors, evidence demonstrates limited additional benefit to increasing RET intensity. Indeed, low-to-moderate intensity RET (30–69% of 1RM) exerts similar improvements in blood pressure (Cornelissen and Smart, 2013), and blood lipid profiles (Lira et al., 2010; Sheikholeslami Vatani et al., 2011) than high-intensity RET (≥70% of 1RM). Thus, contrary to popular belief, we argue that low-to moderate intensity RET (30–69% of 1RM) is safe and effective even in individuals with CVD or at risk for developing CVD.

## Resistance Exercise Training and Cancer

Cancer is a leading cause of morbidity and mortality with approximately 14 million new cases and 9.6 million annual cancer-related deaths worldwide (World Health Organization, Cancer Facts, 2018). Many of these cancer diagnoses share risk factors linked to T2D and CVD and are associated with a sedentary lifestyle (Vainio et al., 2002). In support of this assertion, a wealth of data demonstrate that regular physical activity is associated with a reduced risk of developing cancer, dying from cancer, and improving cancer prognosis (Keum et al., 2016; Moore et al., 2016).

Using data derived from the Health Survey for England and the Scottish Health Survey consisting of 80,000 adults aged >30 years, Stamatakis et al. (2018) demonstrated that adhering to guideline advice to perform RET (at least two times per week) was associated with a 34% reduced risk for cancer mortality; whereas adhering to the AET guidelines provided no statistical benefit. Moreover, cancer survivors who participated in RET at least once per week had a 33% reduction in all-cause mortality (Hardee et al., 2014). A recent comprehensive review conducted by Cormie et al. (2017) demonstrated that regular performance of both RET and AET following the diagnosis of cancer had a protective effect on cancer-specific mortality, cancer recurrence, and all-cause mortality. These observations would be expected, given that muscle mass and strength are inversely associated with cancer mortality (Ruiz et al., 2009; Bennie et al., 2016). Although the aforementioned studies are observational and causation cannot be inferred, together they provide support for the hypothesis that regular performance of RET reduces cancer risk, cancer mortality, and cancer recurrence. Incorporating RET into a combined activity program appears to have complimentary effects on factors related to cancer development.

Dieli-Conwright et al. (2018) demonstrated that following the American College of Sports Medicine/American Cancer Society exercise guidelines for 16 weeks (150 min of AET, and 2–3 sessions of RET/week) in overweight or obese breast cancer survivors improved all components of metabolic syndrome – a comorbid condition prevalent in cancer survivors following treatment that increases the risk for cancer recurrence (Russo et al., 2008) and cancer-specific mortality (Pasanisi et al., 2006). While this work supports the utility of performing both AET and RET in reducing incident and recurrent cancer risk, future randomized controlled trials are warranted to identify which exercise modality (independently) is most effective in this regard.

RET also alleviates patients of some of the unwanted side effects associated with cancer treatment. Current therapeutic approaches (i.e., chemotherapy, radiation therapy, androgen deprivation therapy for prostate cancer) for cancer exacerbate the loss of skeletal muscle mass and strength in patients. Importantly, these adaptions have negative implications for vital clinical endpoints including cancer mortality (Ruiz et al., 2009; Bennie et al., 2016), disease progression, and therapeutic complications (dose-limiting toxicity; Prado et al., 2008). Whole-body, progressive RET (2–4 sets at 60–70% 1RM) can preserve muscle mass and strength in patients with prostate cancer undergoing androgen deprivation therapy (Galvao et al., 2010) or radiation therapy (Segal et al., 2009). A recent meta-analysis in 1200 men with prostate cancer showed that regular RET improved muscular strength, body composition, and 400-m walking performance (Keilani et al., 2017). Importantly, 24 weeks of RET resulted in greater improvements in triglycerides, body fat, and quality of life than AET (cycle ergometer/treadmill/elliptical for 45 min at 60–75% VO_2max_) during radiation therapy (Segal et al., 2009). Exciting data from the Supervised Trial of Aerobic Versus Resistance Training (START) trial demonstrated that whole-body, progressive RET (3 sets at 60–70% 1RM) improved lean body mass, strength, fatigue, and chemotherapy completion rate in breast cancer survivors receiving adjuvant treatment, whereas there was no difference between AET (cycle ergometer/treadmill/elliptical for 45 min at 60–80% VO_2max_) and usual care (Courneya et al., 2007; Adams et al., 2016). In a recent meta-analysis including 11 randomized controlled trials and 1,167 participants (74% women) receiving treatment for various cancers, regular performance of RET led to improvements in lean body mass, strength, and whole-body fat mass (Strasser et al., 2013). These findings are clinically relevant, given that increased adiposity – and the concomitant increase in inflammatory status – is prevalent following cancer treatment and can negatively impact cancer prognosis and increase the risk of recurrence (Vance et al., 2011). Furthermore, the beneficial effects of RET were augmented when RET interventions were of low-to-moderate intensity (≤69% 1RM), which may be more appealing for cancer patients who are unable to – due to comorbidities – lift weights at a high relative intensity (Strasser et al., 2013).

Several biological mechanisms have been proposed to mediate the protective effects of RET on cancer risk and prognosis. RET improves indices of insulin sensitivity, body composition (Strasser et al., 2013), immune function (Hagstrom et al., 2016), sex hormone profile (Ennour-Idrissi et al., 2015; Dieli-Conwright et al., 2018), and markers of inflammation (Strasser et al., 2012; Schmidt et al., 2016; Winters-Stone et al., 2018), all of which are factors hypothesized to contribute to cancer risk and progression (McTiernan, 2008). Recently, skeletal muscle has been recognized as an endocrine organ capable of releasing small peptides into the bloodstream (collectively referred to as myokines), which can exert anti-inflammatory and insulin-sensitizing systemic effects on distant tissues. Given the tight relationship between obesity, insulin resistance, and inflammation with cancer risk and prognosis (Barone et al., 2008; Deng et al., 2016), there is potential for the biological support of exercise-induced myokine secretion in anticancer progression. Exciting data from Pedersen et al. (2016) demonstrate that voluntary wheel running reduced tumor volume by approximately 60% in tumor-bearing C57BL/6 mice. Reductions in tumor volume were associated with natural killer cell infiltration into the tumors, which was dependent upon the release of interleukin-6 (IL-6) from contracting skeletal muscle (Pedersen et al., 2016). In fact, the entire process of IL-6 release from contracting skeletal muscles appeared to be unique as intravenous injections of IL-6 failed to reduce tumor growth (Pedersen et al., 2016). Although the results of Pedersen et al. (2016) demonstrate that contracting skeletal muscles are capable of naturally manufacturing molecules with anti-tumorigenic properties, far less is known regarding the role of RET on myokine release. Given that myokine release in humans is a process dependent upon the contraction of skeletal muscle (Hojman et al., 2018), we hypothesize that RET would lead to a similar increase in myokine secretion as AET. Thus, the relationship between RET and myokine release in combatting malignant tumors warrants investigation.

Similar to AET, there is a role for RET in reducing cancer risk, cancer recurrence, cancer mortality, and improving prognosis during adjuvant therapies. Given that cancer has surpassed CVD as the leading cause of death in several developed countries (Tanday, 2016), these observations are of great importance. Although the importance of RET for breast cancer and prostate cancer is becoming apparent, the effects of RET on other cancer types are equivocal, and warrant further investigation. Future work should be focused upon unraveling the optimal dose, intensity, and mechanisms specific to RET-induced cancer benefits.

## Resistance Exercise Training Recommendations for Reducing Age-Related Chronic Disease Risk

**Figure 1:**
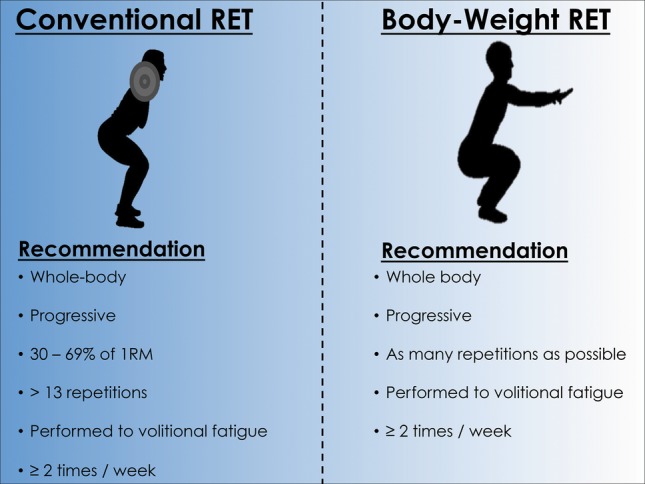
Evidence-based resistance exercise training recommendations for reducing age-related chronic disease risk. Abbreviations: 1RM, 1 repetition maximum.

The wide-ranging health benefits of regular RET are well established; however, adherence to RET in older adults remains low, and the most commonly cited barriers to participation of RET are: (1) risk of injury (from lifting heavy relative loads) and (2) required access to a gym facility (Burton et al., 2017). However, utilizing one’s own body weight as resistance, or light-to-moderate relative loads (30–69% of 1RM) is just as effective as lifting heavy relative loads (≥70% of 1RM) for exerting health benefits (Lira et al., 2010; Lustosa et al., 2011; Sheikholeslami Vatani et al., 2011; Cornelissen and Smart, 2013; Strasser et al., 2013; Csapo and Alegre, 2016; Yang et al., 2017; Stamatakis et al., 2018). Cognizant of these findings, RET recommendations have been formulated, which may aid older adults in adhering to and thus reducing chronic disease risk (Figure 1). We suggest that exercise volume is more salient than exercise intensity in mediating the positive adaptations discussed herein, and that as long as RET is performed to volitional fatigue, older adults can reap the health benefits of RET.

## Resistance Exercise Training: From a Supporting to a Starring Role?

**Figure 2:**
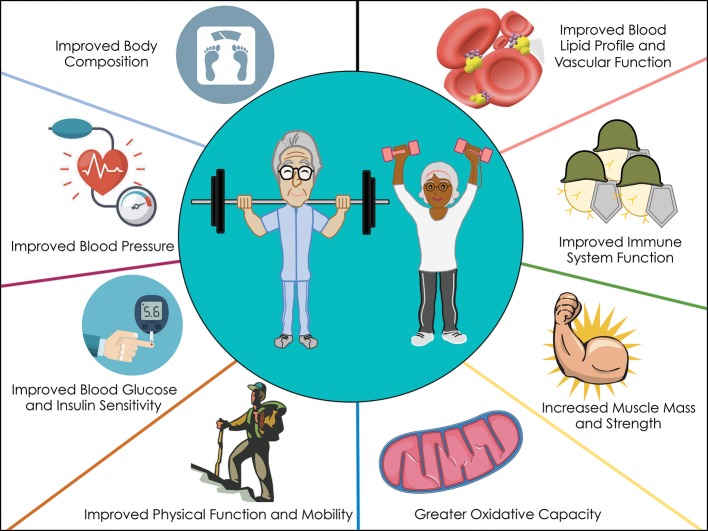
Proposed mechanisms whereby RET influences chronic disease risk.

The evidence presented in this review demonstrates the beneficial effects of RET on reducing chronic disease risk (mobility disability, T2D, CVD, and cancer) in older adults (Figure 2). Regular performance of RET improves muscle mass, strength, and function, and can have direct effects on the primary prevention of a number of chronic diseases. On the basis of the evidence we have highlighted, RET-induced benefits in chronic disease risk are equivalent if not superior in magnitude as AET (Table 1). Nonetheless, a number of agencies endorse performing 150 min of AET per week to mitigate age-related chronic disease risk, whereas the role of RET on overall health is typically underappreciated. Furthermore, only 2.4% of older adults achieve this AET recommendation (Troiano et al., 2008), and this may be due in part to the guidelines including intensities or volumes potentially unreachable for older adults limited by many comorbidities. Based on the evidence presented in this narrative review, we propose that RET may serve as *“another tool in the toolbox”* for older adults to remain physically active and combat chronic disease risk. We do acknowledge that some knowledge gaps exist such as the optimal dose and intensity of RET required to exert health benefits and clinical trial evidence showing head-to-head comparisons with AET, and further investigations are needed.

**Table 1:** Age-related changes in risk factors for chronic disease and the adaptive responses to aerobic exercise training and resistance exercise training.

Adaptations	Aging	AET	RET
**Whole-body adaptations**			
Muscle strength	↓	↔ (Grgic et al., 2018)	↑↑ (Cadore et al., 2013)
Muscle mass	↓	↔ (Grgic et al., 2018)	↑↑ (Peterson et al., 2011)
Bone mineral density	↓	↔ (Villareal et al., 2017)	↑ (Villareal et al., 2017)
VO_2peak_	↓	↑↑ (Bienso et al., 2015)	↑ (Bienso et al., 2015)
Physical function	↓	↑ (Jadczak et al., 2018)	↑ (Jadczak et al., 2018)
**Type II diabetes**			
Risk	↑	↓ (Knowler et al., 2002)	↓ (Grontved et al., 2012)
Glycemic control	↓	↑ (Bienso et al., 2015)	↑ (Bienso et al., 2015)
Insulin signaling	↓	↑ (Bienso et al., 2015)	↑ (Bienso et al., 2015)
Oxidative capacity	↓	↑ (Jubrias et al., 2001)	↑ (Jubrias et al., 2001)
**Cardiovascular disease**			
Risk	↑	↓ (Tanasescu et al., 2002)	↓ (Tanasescu et al., 2002)
Blood pressure	↑	↔ (Cornelissen and Smart, 2013)	↓ (Cornelissen and Smart, 2013)
Blood lipids			
High-density lipoprotein	↓	↑ (Yang et al., 2014)	↑ (Yang et al., 2014)
Low-density lipoprotein	↑	↓ (Yang et al., 2014)	↓ (Yang et al., 2014)
Cholesterol	↑	↓ (Yang et al., 2014)	↓ (Yang et al., 2014)
Triglycerides	↑	↓ (Yang et al., 2014)	↓ (Yang et al., 2014)
**Cancer**			
Incident Risk	↑	↓ (Keum et al., 2016)	↓ (Keum et al., 2016)
Risk of recurrence	↑	↓ (Dieli-Conwright et al., 2018)	↓ (Dieli-Conwright et al., 2018)
Quality of life	N/A	↑ (Segal et al., 2009)	↑↑ (Segal et al., 2009)
Therapy completion rate	N/A	↔ (Courneya et al., 2007)	↑ (Courneya et al., 2007)
Immune function	↓	↑ (McTiernan, 2008)	↑ (Hagstrom et al., 2016)
Inflammation	↑	↓ (McTiernan, 2008)	↓ (Strasser et al., 2012)

↑ *Indicates an increasing effect on the parameter,* ↓ *indicates a decreasing effect on the parameter, and ↔ indicates no effect on the parameter. The number of arrows denotes the magnitude of effect. Abbreviations: N/A, not available.*

## Author Contributions

JCM, TS, and SMP wrote the initial draft of the manuscript. All authors edited and approved the final version of the manuscript and agree to be accountable for all aspects of the work. All persons designated as authors qualify for authorship, and all those who qualify for authorship are listed.

### Conflict of Interest Statement

The authors declare that the research was conducted in the absence of any commercial or financial relationships that could be construed as a potential conflict of interest.
